# The Impact of COVID-19 Outbreak on Syncope Units Activities in Italy: A Report from the Italian Multidisciplinary Working Group on Syncope (GIMSI)

**DOI:** 10.3390/ijerph18179194

**Published:** 2021-08-31

**Authors:** Vincenzo Russo, Giulio Boggian, Maria Giulia Bolognesi, Domenico Maria Carretta, Simone Cencetti, Domenica De Laura, Enzo Hrovatin, Paolo Pastori, Caterina Tomaselli, Erika Parente, Martina Rafanelli, Andrea Ungar, on behalf of COVID-19 GIMSI Study Group

**Affiliations:** 1 Cardiology and Syncope Unit, Department of Medical Translational Sciences, University of Campania “Luigi Vanvitelli”—Monaldi Hospital, 80131 Naples, Italy; parente-erika@libero.it; 2 Internal Medicine and Syncope Unit, Bentivoglio Hospital, Health Authority Bologna, 40053 Bologna, Italy; g.boggian@ausl.bologna.it; 3 Cardiology and Syncope Unit, Guglielmo da Saliceto Hospital, 29121 Piacenza, Italy; m.bolognesi@ausl.pc.it; 4 Cardiology and Syncope Unit, Giovanni XXIII Hospital, 70126 Bari, Italy; carrettacardiologia@gmail.com; 5 Internal Medicine and Syncope Unit, Piero Palagi Hospital, Health Authority Toscana Centro, 50122 Firenze, Italy; simone.cencetti@uslcentro.toscana.it; 6 Cardiology and Syncope Unit, San Paolo Hospital, 70123 Bari, Italy; ndelaura@libero.it; 7 Cardiology and Syncope Unit, Monfalcone Hospital, Health Authority Giuliano Isontina (GO), 34074 Monfalcone, Italy; e.hrovatin@tin.it; 8 Cardiology and Syncope Unit, Fidenza Hospital, Health Authority Parma, 43036 Fidenza, Italy; ppastori@ausl.pr.it; 9 Cardiology and Syncope Unit, Annunziata Hospital, 87100 Cosenza, Italy; caterina.tomaselli@tiscali.it; 10 Geriatrics and Intensive Care Unit, Hypertension Centre—Syncope Unit University of Florence and Careggi Hospital, 50134 Florence, Italy; martina.rafanelli@unifi.it (M.R.); andrea.ungar@unifi.it (A.U.)

**Keywords:** syncope, syncope unit, COVID-19, outbreak, lockdown, clinical activities, cardiac invasive procedure

## Abstract

The aim of our study was to evaluate the impact of the COVID-19 outbreak on Syncope Units (SUs) Activities in Italy. **Methods:** Data about types of SU activities and admissions were obtained from 10 SUs throughout Italy, certified by the Italian Multidisciplinary Working Group on Syncope (GIMSI), from 10 March 2020 to 31 December 2020 and compared with the same time frame in 2019. **Results:** A remarkable reduction in overall non-invasive diagnostic tests (−67%; *p* < 0.001) and cardiac invasive procedure. Elective cardiac pacing procedures disclosed a significant decrease (−62.7%; *p* < 0.001); conversely, the decrease of urgent procedures was not significant (−50%; *p =* 0.08). There was a significantly increased rate of patients who underwent both telemedicine follow-up visits (+225%, *p* < 0.001) and cardiac implantable electronic devices (CIEDs) remote monitoring follow-up visits (+100%; *p* < 0.001). **Conclusion:** The COVID-19 outbreak was associated with a remarkable decrease in all clinical activities of Syncope Units in Italy, including both non-invasive tests and cardiac invasive procedures; conversely, a significant increase in telehealth activities was shown.

## 1. Introduction

Severe acute respiratory syndrome coronavirus 2 (SARS-CoV-2) is a highly pathogenic human coronavirus recognized as the cause of coronavirus disease 2019 (COVID-19) [1]. The outbreak started in China and rapidly spread worldwide, reaching devastating pandemic proportions with alarming morbidity and mortality [2]. Italy was among the countries majorly hit by COVID-19, with more than 4,241,760 laboratory-confirmed cases by 12 July 2021 and more than 126,924 deaths [3]. Following the COVID-19 outbreak, the Italian government adopted strict rules characterized by a national lockdown, from 10 March to 4 May 2020, a partial nationwide movement restriction, mandatory mask use and social distancing as an attempt to contain the virus diffusion [4]; consequently, some changes in the pattern of hospital activities or admissions for cardiovascular conditions have been observed [5,6,7,8,9,10]. A Syncope Unit (SU) is a facility featuring a standardized approach to the diagnosis and management of transient lack of consciousness (TLOC) and related symptoms, with dedicated staff and access to appropriate diagnostics and therapies [11]. The aim of our study was to evaluate the impact of the COVID-19 outbreak on Syncope Units activities in Italy.

## 2. Materials and Methods

This is a retrospective multicenter observational study. Data about syncope unit activities including clinical and instrumental non-invasive evaluations, interventional cardiac procedures, type of SU admission and diagnosis were obtained from 10 Syncope Units, certified by the Italian Multidisciplinary Working Group on Syncope (GIMSI) throughout Italy, from 10 March 2020 to 31 December 2020 and compared within the same time frame in 2019. Assessed instrumental non-invasive examinations were all tests currently used for syncope evaluation according to the current guidelines [11]. Assessed cardiac pacing procedures were pacemaker (PM), implantable cardiac defibrillator (ICD) and implantable loop recorder (ILR) implantation. Moreover, the number of patients who underwent telemedicine follow-up visits and cardiac implantable electronic devices (CIEDs) remote monitoring follow-up visits were assessed. The admission to SU services was differentiated into outpatient visits, in-hospital consultancies and emergency department consultancies. The study was approved by the Local Ethics Committee and was in accordance with 1976 Declaration of Helsinki and its later amendments. All data are presented as either number and percentage, in the case of categorical variables, or median and interquartile range (IQR) for what concerns continuous variables, after appropriately testing their distribution by the Shapiro–Wilk and Kolmogorov–Smirnov goodness-of-fit tests. Differences between variables were either assessed by the chi-square test for categorical variables or the nonparametric Mann–Whitney U test. In addition, an appropriate Generalized Linear Model with Log-Linear Poisson regression for modeling count data was implemented to compute incidence rate ratios for all single procedures (reduction and increase rates, respectively). A *p*-value less than 0.05 was considered statistically significant. All analyses were performed by SPSS Software, Version 24 (IBM, Armonk, NY, USA) and STATA 14.0 software (StataCorp. 2015. College Station, TX, USA: StataCorp LP).

## 3. Results

**Figure 1:**
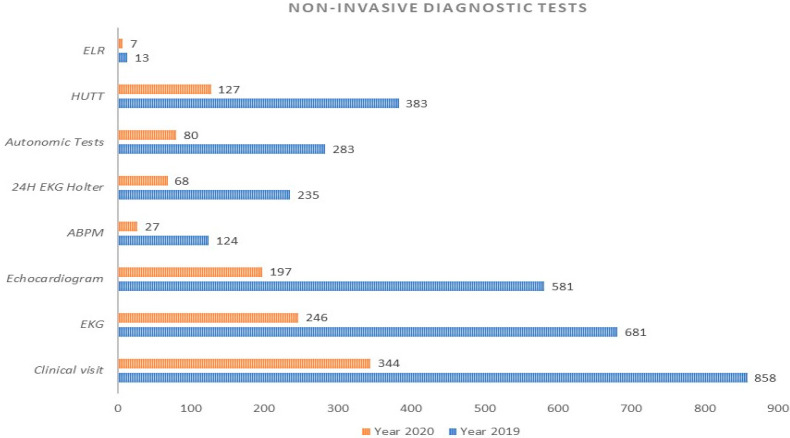
Number and type of non-invasive diagnostic tests performed at syncope units during 2019 and 2020 study periods. ELR: external lop recorder; HUTT: head up tilt test; EKG: electrocardiogram; ABPM: ambulatory blood pressure monitoring.

One thousand two hundred and seventy-five patients who were referred to SUs for the management of TLOC and related symptoms over the two observation periods were enrolled in the study, of which 377 were during the national lockdown for COVID-19 outbreak and 898 during the same time period in 2019. Patients treated during the COVID-19 lockdown were significantly younger than those treated in 2019 [median age 63 years (IQR: 46–80) vs. 70 years (IQR: 46–80) vs. *p* = 0.04], whilst both subgroups were equally distributed for sex. The clinical visits for the initial TLOC evaluation were 858 in 2019 vs. 344 in 2020, with a reduction rate of −59% (*p* = 0.001). Overall non-invasive diagnostic tests were 2.300 in 2019 vs. 752 in 2020, with a reduction rate of −67% (*p* < 0.001). The type and number of diagnostic tests are graphically represented in Figure 1.

**Figure 2:**
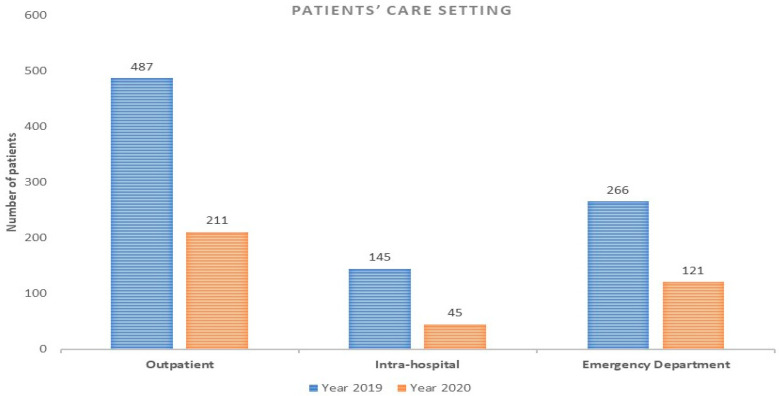
Patients’ care setting during 2019 and 2020 study periods.

As for the type of patients’ setting care, the highest reduction rate was observed for intra-hospital activities (reduction rate: −69%; *p* < 0.008), followed by a remarkable reduction in both outpatient (reduction rate: −57%; *p* < 0.002) and emergency department activities (reduction rate: −54%*; p <* 0.008) (Figure 2).

**Figure 3:**
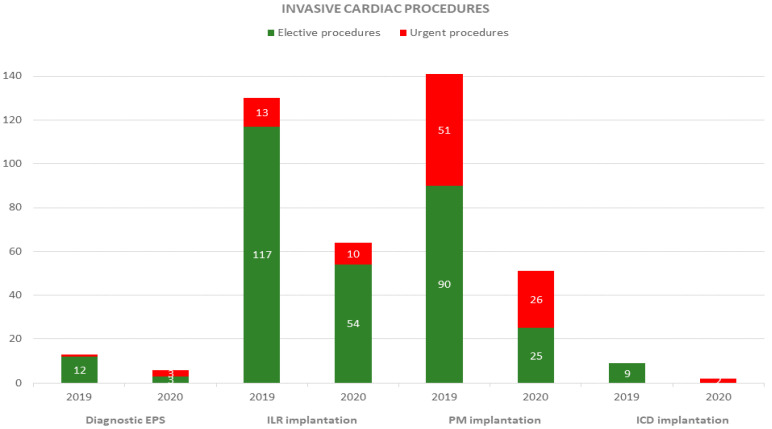
Number and type (elective or urgent) of invasive cardiac procedures during 2019 and 2020 study periods.EPS: electrophysiological study; ILR: implantable loop recorder; PM: pacemaker; ICD: implantable cardioverted defibrillator.

Overall invasive cardiac procedures were 293 in 2019 vs. 124 in 2020, with a reduction rate of −57.7% (*p* < 0.001). There was a remarkable reduction in both ILR (reduction rate: −50%; *p* < 0.009) and PM implantation (reduction rate: −63%; *p* = 0.008). Elective cardiac pacing procedures disclosed a significant decrease (228 in 2019 vs. 85 in 2020; reduction rate: −62.7%; *p* <0.001); conversely, the decrease of urgent procedures was not significant (65 in 2019 vs. 39 in 2020; reduction rate: −40%; *p* = 0.08) (Figure 3).

**Figure 4:**
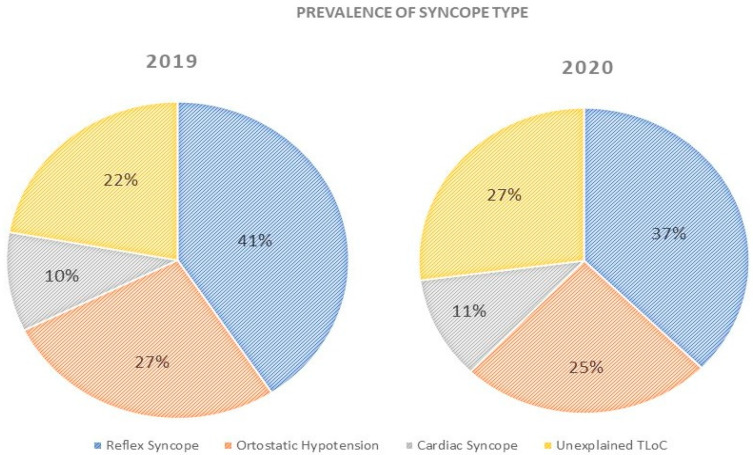
Prevalence of syncope type during 2019 and 2020 study periods.

Despite a significant reduction rate in the diagnosis of all types of syncope, no differences of prevalence according to the type were shown across the two observation periods (Figure 4).

**Figure 5:**
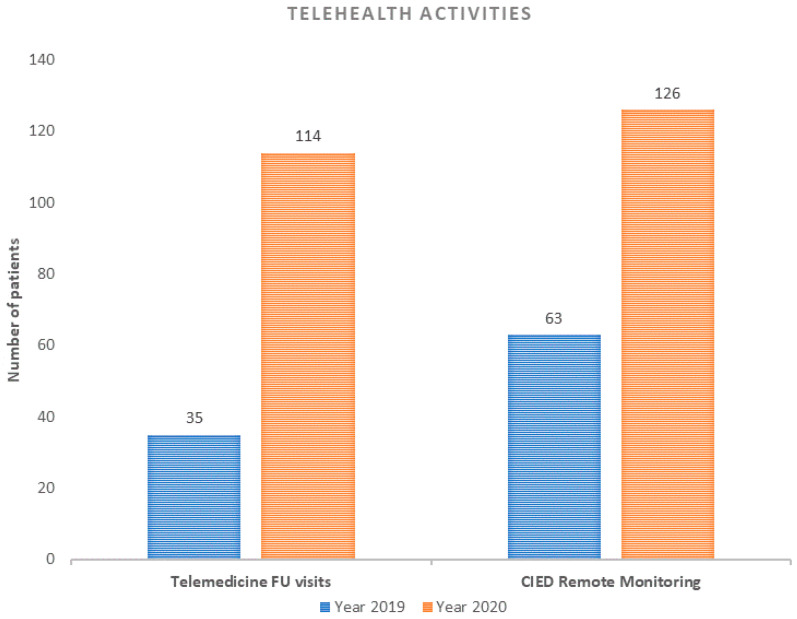
Number and type of telehealth activities during 2019 and 2020 study periods. FU: follow-up; CIED: cardiac implantable electronic device.

There was a significant increased rate of patients who underwent both telemedicine follow-up visits (35 in 2019 vs. 114 in 2020; increase rate: +225%, *p* < 0.001) and CIED remote monitoring follow-up visits (63 in 2019 vs. 126 in 2020; increase rate: +100%; *p* < 0.001) (Figure 5). Population-specific data are shown in Table 1.

## 4. Discussion

Our findings suggest that the COVID-19 lockdown was associated with a significant reduction rate in all the clinical activities of Syncope Units in Italy, including both non-invasive tests and cardiac invasive procedures. The remarkable changes in the SUs activities were more likely because of the reduction of patients referred from other hospital wards, which were converted into COVID-19 care centers; moreover, we noticed a reduction of outpatient accesses to SUs, mainly due to the Italian government’s measures to contain SARS-CoV-2 diffusion. The reduction of the emergency department admission rate for patients in need of an SU specialist evaluation might be explained by the fear of acquiring COVID-19 infection once hospitalized.

Our evidences support and enhance the data which described the reduction in the hospitalization rate for percutaneous coronary intervention [5,6] and cardiac pacing procedures [7,8,9,10] during the COVID-19 pandemic; moreover, our results suggest the hypothesis that the undiagnosed TLOC episodes may have contributed to the increase in non-COVID-19 out-of-hospital mortality in Italy [11].

The remarkable increasing use of remote monitoring for the follow-up of CIEDs recipients and the increasing telemedicine follow-up visits for patients who experienced TLOC and related symptoms during the COVID-19 lockdown suggest that telehealth helped us to assure continuous care, reducing disease exposure for staff and physicians [12,13]. In this sense, telehealth may also represent a tool to be further used in the future [14,15]. The lack of an adequate reimbursement and the absence of sharing standards for CIEDs’ remote monitoring or medical teleconsultation represent the most important reported barriers to the implementation of telehealth tools in the clinical practice [16].

## 5. Conclusions

The COVID-19 outbreak caused a remarkable reduction of patients who were referred to SUs for the management of TLOC and related symptoms. This evidence suggests the hypothesis that TLOC episodes may have contributed to the increase in non-COVID-19 out-of-hospital mortality in Italy.

## Figures and Tables

**Table 1:** Anthropometric characteristics and differences in SUs activities during 2019 and 2020 study periods.

Parameter	Year 2019 (*n* = 898)	Year 2020 (*n* = 377)	RR/RI	*p*
Age (years), median [IQR]	70 [46–80]	63 [46–80]		0.04
Male Sex, *n* (%)	472 (52, 5)	194 (51, 5)		0.7
Non-invasive tests				
Clinical visit, *n* (%)	858 (95, 5)	344 (91, 25)	−59%	<0.001
EKG, *n* (%)	681 (75, 8)	246 (65, 25)	−49%	<0.001
Echocardiogram, *n* (%)	581 (64, 7)	197 (52, 25)	−66%	<0.001
ABPM, *n* (%)	124 (13, 3)	27 (7, 1)	−78%	<0.001
24H EKG Holter, *n* (%)	235 (26, 17)	68 (18)	−71%	<0.001
Autonomic Tests, *n* (%)	283 (31, 51)	80 (21)	−61%	<0.001
HUTT, *n* (%)	383 (42, 65)	127 (33, 7)	−71%	<0.001
ELR, *n* (%)	13 (1, 4)	7 (1, 85)	−46%	0.07
Patients’ Care Setting				
Outpatient, *n* (%)	487 (54, 23)	211 (55, 96)	−57%	0.002
Intra-hospital, *n* (%)	145 (16, 15)	45 (11, 94)	−69%	0.008
Emergency Department, *n* (%)	266 (29, 62)	121 (32, 09)	−54%	0.008
Invasive Procedures				
Diagnostic EPS, *n* (%)	13 (1, 4)	6 (1, 6)	−53%	0.07
ILR implantation, *n* (%)	130 (14, 5)	65 (17, 24)	−50%	0.009
PM implantation, *n* (%)	141 (15, 7)	51 (13, 5)	−63%	0.008
ICD implantation, *n* (%)	9 (1)	2 (0, 53)	−77%	0.08
Timing of Invasive Procedures				
Elective procedures, *n* (%)	228 (25, 38)	85 (22, 54)	−62.7%	<0.001
Urgent procedures, *n* (%)	65 (7, 23)	39 (10, 34)	−40%	0.08
Telehealth activities				
Telemedicine FU visits, *n* (%)	35 (3, 9)	114 (30, 2)	+225%	<0.001
CIED Remote Monitoring, *n* (%)	63 (7, 01)	126 (33, 4)	+100%	<0.001

EKG: electrocardiogram; ABPM: ambulatory blood pressure monitoring: HUTT: head up tilt test; ELR: external loop recorder; EPS: electrophysiological study; ILR: implantable loop recorder; PM: pacemaker; ICD: implantable cardioverted defibrillator; FU: follow-up: CIED: cardiac implantable electronic device.

## Data Availability

The data presented in this study are available on request from the corresponding author.
